# Background Speech Effects on Sentence Processing during Reading: An Eye Movement Study

**DOI:** 10.1371/journal.pone.0152133

**Published:** 2016-03-22

**Authors:** Jukka Hyönä, Miia Ekholm

**Affiliations:** 1 Department of Psychology, University of Turku, Turku, Finland; 2 Stockholm South General Hospital (Södersjukhuset), Stockholm, Sweden; University of Leicester, UNITED KINGDOM

## Abstract

Effects of background speech on reading were examined by playing aloud different types of background speech, while participants read long, syntactically complex and less complex sentences embedded in text. Readers’ eye movement patterns were used to study online sentence comprehension. Effects of background speech were primarily seen in rereading time. In Experiment 1, foreign-language background speech did not disrupt sentence processing. Experiment 2 demonstrated robust disruption in reading as a result of semantically and syntactically anomalous scrambled background speech preserving normal sentence-like intonation. Scrambled speech that was constructed from the text to-be read did not disrupt reading more than scrambled speech constructed from a different, semantically unrelated text. Experiment 3 showed that scrambled speech exacerbated the syntactic complexity effect more than coherent background speech, which also interfered with reading. Experiment 4 demonstrated that both semantically and syntactically anomalous speech produced no more disruption in reading than semantically anomalous but syntactically correct background speech. The pattern of results is best explained by a semantic account that stresses the importance of similarity in semantic processing, but not similarity in semantic content, between the reading task and background speech.

## Introduction

Reading is done in many different physical environments. It may be done during a quiet evening lying on a couch undisturbed by any external sources of visual or auditory information. It may also be done in a noisy environment, such as a crowded cafeteria or a busy train or subway couch, where a lively discussion or a phone conversation may be heard in the background. Many people prefer to read in silence and find noisy environments distracting and disturbing for reading.

The present study was designed to examine possible disruption effects by background speech on online text processing. Online processing was measured by registering readers’ eye movements while they read texts at their own pace. There is now ample evidence [1] that eye-tracking is a sensitive and reliable measure to study different aspects of on-line language processing, from word recognition through syntactic parsing to discourse processing. Thus, the method provides a real-time protocol of the comprehension process as it evolves through time and space.

To our knowledge, the study of Cauchard et al [2] is the only one investigating disruption effects on the online reading process. They found overall slow-down in reading short text passages when it was performed in the presence of background speech (a radio talk show). A significant portion of the slow-down was due to rereading fixations made during the first-pass reading of sentences and also due to later look-back fixations launched to the target sentences from subsequent text sentences.

Although studies on the effects of background speech on the online reading process are largely lacking, there is rather extensive literature on disruption effects by background speech on language comprehension, measured after reading; for effects on proofreading, see [3–5].

Martin et al. [6] assessed the end result of the comprehension process by asking questions about the text contents after reading. The study showed that when meaningful material (prose or random words) was presented in the background during reading, comprehension scores were poorer than with non-meaningful background stimuli. They concluded that reading comprehension is disrupted by irrelevant background stimuli only when the distracting stimuli call upon the same processing mechanisms and representations (i.e., semantic processing) than the primary task. Oswald et al. [7] studied the effects of irrelevant meaningful and meaningless speech in a task, in which subjects were asked questions about single sentences. The study showed that both types of irrelevant speech brought about a disruption effect in comparison to the silent condition, but the effect was significantly greater for meaningful speech. The study provides evidence that irrelevant speech is capable of interfering with semantic processing during sentence comprehension. Sörqvist et al. [8] found that participants made more errors in answering to reading comprehension questions when reading short texts for comprehension when an auditorily presented story was played in the background, compared to silence. On the other hand, using error rates and decision times in making judgments of the semantic acceptability of syntactically complex and less complex sentences Boyle and Coltheart [9] did not observe irrelevant speech or other vocal sounds to significantly impair reading comprehension. Finally Robison and Unsworth [10] found no difference between the silent reading condition and the bar noise (voices, music, etc.) condition in the accuracy in answering to comprehension question.

Effects of background speech have also been studied on text memory. Banbury and Berry [11] found robust effects of background speech (radio program or recorded office noise with speech) on serial recall of a short text passage. They also found meaningless background speech (Greek speech presented to native English speakers) to disrupt text memory. Sörqvist et al. [12] found memory for prose, measured with comprehension questions asked after reading, to be poorer when irrelevant speech was played in the background, compared to silence. Similar results were obtained by Sörqvist [13] when the background speech condition was compared to aircraft noise. On the other hand, Haapakangas et al. [14] found no effect of background speech (excerpts from different radio talk shows mixed with each other) on free recall of the contents of a relatively long expository text.

Another goal of the present study was to shed light on the nature of irrelevant background speech effects on online text comprehension. Theorizing about the nature of interference in reading comprehension due to irrelevant speech has resulted in several alternative accounts. One possibility is offered by the seminal model of working memory put forth by Baddeley [15–16]. According to his theory, verbal materials are temporarily buffered as verbatim representations in the phonological store. All speech inputs, including task-irrelevant background speech, gain obligatory access to the phonological store, whereas written materials need to be recoded into subvocal speech by the rehearsal mechanism to yield access to the phonological store. Thus, when phonological representations constructed and retained in the phonological loop system (phonological store + rehearsal mechanism) for the written text coexists with irrelevant speech having automatic access to the store, interference in written language comprehension will result by the irrelevant speech corrupting the phonological representations built for the written text. According to the model, all speech-like inputs will lead to disruption effects regardless of the meaningfulness of the irrelevant speech (however, it does not deny the possibility that meaningful speech may lead to greater disruption than meaningless speech). Gathercole and Baddeley [17] further propose that the phonological loop is particularly pertinent in comprehending long and syntactically complex sentences.

Using in-depth analyses of neurological patients as evidence, Martin and colleagues [18–19] have proposed a multiple-components view of verbal working memory. According to their model, temporary retention of verbal materials requires separate capacities for phonological, semantic and syntactic information. If so, irrelevant speech may disrupt phonological, semantic or syntactic processing and temporary retention. Martin and colleagues emphasize the importance of semantic capacities. More specifically, they [6] proposed that the interference effect in reading comprehension due irrelevant background stimuli is semantic in nature. They suggest that when the distracting stimuli call upon the same semantic processing mechanisms and representations as the primary reading task, reading comprehension is disrupted. Jones et al. [4] offered a similar explanation that makes reference to two streams of information both calling for analysis at the level of meaning. The same idea is put forth by the interference-by process account of auditory distraction proposed by Marsh et al. [20–21]. These views contradict with that of Baddeley in that they do not predict interference by meaningless speech (e.g., foreign language speech), whereas Baddeley’s model predicts all speech input to interfere with written language comprehension. As reviewed above, previous research on irrelevant speech effects on reading comprehension indeed suggests that meaningful speech may be more disruptive than meaningless speech [4–7].

Another possibility, not directly addressed by prior studies, is that interference by irrelevant speech is syntactic in nature. This would be in line with the multiple-components view of Martin and colleagues. Such hypothesis may also be motivated on the basis of the separate-sentence-interpretation-resource (SSIR) model of verbal working memory by Caplan and Waters [22]. The model assumes a separate sentence interpretation module, which is equipped with an independent resource pool that is responsible for syntactic processing. The system is assumed to be modular in the sense that it processes linguistic input in a reasonably automatic fashion. On the assumption that this sentence interpretation unit automatically processes all available verbal stimuli (analogously to the phonological loop), its workings may be interfered by feeding into the module task-irrelevant verbal input that cannot be syntactically parsed or is difficult to parse. Thus, although not explicitly stated by Caplan and Waters, the SSIR model appears to predict an interference effect in reading as a result of concurrently presented speech, particularly when it is syntactically illegal. It may be further predicted that the interference effect is more pronounced when reading syntactically complex than less complex sentences. This is because two types of difficult-to-process verbal stimuli, syntactically complex sentences to be read and auditorily presented syntactically illegal sentences, will be fed into the sentence interpretation module.

As mentioned above, the present study had two main goals. First, we wanted to examine whether background speech has adverse effects on online written language processing. Second, we aimed to test whether the interference is primarily phonological, semantic or syntactic in nature. Four eye-tracking experiments were conducted, where participants read texts for comprehension while different types of irrelevant speech were played in the background. Effects of background speech were examined for long target sentences, for which syntactic complexity was also manipulated.

In Experiment 1, meaningful (native language) and non-meaningful (foreign language) speech was played in the background. It was carried out to test the prediction derived from the phonological loop model [15], which assumes all kind of verbal input, regardless of its meaningfulness to interfere with reading comprehension. In the subsequent experiments, we interfered with syntactic and semantic processing of the to-be-read text by presenting in the background semantically and/or syntactically anomalous speech. For Experiment 2, we prepared two types of syntactically and semantically anomalous speech outputs by scrambling the order of words of two coherent texts and by reading them aloud with normal sentence intonation. In the Scrambled-Same condition, the scrambled speech was created by scrambling the order of words of the same text the participants were reading, while in the Scrambled-Different condition the speech was created from a different text. The idea here was to test whether shared semantic representations between the text to-be-read and the background speech (i.e., the Scrambled-Same condition) would be particularly disruptive. In Experiment 3, effects of scrambled background speech were directly compared to those of coherent speech. Finally, in Experiment 4 we contrasted a background speech condition that was semantically anomalous but syntactically correct to a condition that was both semantically and syntactically anomalous. The idea behind Experiment 4 was to contrast the syntactic and semantic accounts outlined above. According to the syntactic account adding syntactic anomaly should exacerbate the disruption effect.

According to Gathercole and Baddeley [17], disruption in reading due to irrelevant speech is more likely to manifest when reading is made more difficult. An exactly opposite prediction is recently made by Sörqvist and Marsh [23]. They argue that difficulty in processing increases concentration in the primary task, which in turn shields against distraction by task-irrelevant stimuli. We manipulated reading difficulty by manipulating the syntactic complexity of long, center-embedded relative clause sentences embedded in text. Two types of sentences were formed. In the easier ones, the word order of the main clause complied with the default subject-verb-object (SVO) order in Finnish (the native language of the participants); in the more difficult ones, the word order was syntactically marked (object-verb-subject = OVS, or object-subject-verb = OSV). The difficulty manipulation had two aims. First, it allowed us to examine the contrasting accounts made by Gathercole and Baddeley and Sörqvist and Marsh. Second, the manipulation had particular significance for addressing the syntactic account sketched above.

In Finnish word order is fairly flexible, although the SVO order is by far the most commonly used [24]. Yet, OVS and OSV orders are also possible. Consider Sentence (1), in which the main clause begins with a syntactic object, and the subject appears only after the relative clause. Compare this to Sentence (2), in which the main clause conforms to the default SVO word order.

1. *Pyramidialuetta*, *joka oli tärkeä osa kaupunkia*, *ihmiset kutsuivat nimellä Kuolleiden kaupunki*.
  “The pyramid area that was an important part of the town people called the Town of the Dead.”
2. *Pyramidialue*, *joka oli tärkeä osa kaupunkia*, *muodosti niin sanotun Kuolleiden kaupungin*.
  “The pyramid area that was an important part of the town comprised the so-called Town of the Dead.”

The initial constituent in Sentence (1) (i.e., *pyramidialuetta*) appears in the so-called partitive case, which is one of three case inflections to grammatically mark a clause object in Finnish. The clause subject (i.e., *ihmiset* = people) succeeds the relative clause and appears in the nominative case. On the other hand, in Sentence (2) the initial constituent is the clause subject (morphologically marked as such), which is first followed by the relative clause and then by the verb and the object phrase of the main clause.

Hyönä and Hujanen [25] have demonstrated that sentences beginning with an object are more difficult to parse in Finnish than sentences with the default SVO order. In the readers’ eye fixation records, the processing difficulty was already apparent in the initial encounter with the first constituent, but it was also seen in increased number of reinspective fixations directed back to the initial constituent. That object-fronted clauses indeed induce a processing difficulty has also been demonstrated by MacWhinney and Pléh [26] for Hungarian (a language related and structurally similar to Finnish), by Hemforth et al. [27] for German, and several studies for English [28–30]. Gibson [31] has put forth a theoretical processing model that can account for the aforementioned word order effects (and other syntactic complexity effects).

In addition to the marked word order, the difficulty of the sentence construction in Sentence (1) is exacerbated by the fact that the discourse entity (i.e., ‘pyramid area’) referred to by the initial constituent of the main clause and by the relative pronoun of the embedded clause occupies two different syntactic roles; it is at the same time the object of the main clause and the subject of the relative clause. On the other hand, in Sentence (2) both the initial constituent and the relative pronoun are clause subjects. That this dual-role feature in Sentence (1) indeed produces an additional comprehension difficulty was demonstrated by Kliegl et al. [32] for similarly structured sentences in German; see also [29].

In general, sentence constructions in which part of an uncompleted clause needs to be kept active in working memory while processing another clause [30–31] are instances of sentences whose processing are assumed to require some sort of working memory system. The reader may temporarily store clause fragments in a verbatim form with the help of the phonological loop [9,17] or in some more abstract form. The key point here is that if disruption by irrelevant speech exists in reading the effects are likely to be seen when processing the type of syntactically complex sentence employed in the present study. The pairs of more and less complex sentences were practically identical in length and highly similar in their semantic content. We embedded the target sentences in texts rather than presented them in isolation so that the experimental task would maximally mimic a natural reading situation.

## Experiment 1

In Experiment 1, reading of long sentences embedded in text was tested in three different background speech conditions: in silence, with Italian background speech (non-meaningful to the participants), and with Finnish background speech (meaningful to the participants). Based on the phonological loop model [15], it was hypothesized that if phonological loop is in operation during the comprehension of long, syntactically complex sentences, sentence processing is disrupted by the presence of background speech, in comparison to the silent condition, regardless of the meaningfulness of the background speech (yet, meaningful background speech may disrupt more than meaningless background speech). The model further predicts a more robust syntactic complexity effect during the two irrelevant speech conditions compared to the silent condition. This prediction is based on the assumption that syntactic complexity increases the comprehender’s reliance on the contents of the phonological short-term store [17] and that irrelevant speech corrupts or overwrites its contents so that the verbatim representation of the initial constituent of the main clause will be lost by the time the reader encounters the rest of the main clause. This loss is assumed to be more detrimental to the processing of complex than less complex sentence constructions. On the other hand, the view advocated by Sörqvist and Marsh [23] does not predict such an effect, as processing difficulty is assumed to shield against distraction by task-irrelevant stimuli.

The inclusion of two irrelevant speech conditions makes possible to examine whether the meaningfulness of the background speech is capable of modulating the disruption effect. As reviewed above, a wealth of evidence exists of the effects of meaningfulness of background speech on memory. Yet, as comparable studies on online reading process are largely lacking, it was deemed important to examine it in the context of text processing. According to the phonological loop model, all speech-like stimuli should be disruptive. On the other hand, according to the semantic interference account [6], only meaningful speech will interfere with reading comprehension. A similar prediction may be made on the basis of the above-outlined syntactic account. The syntactic module would process native-language speech but not foreign-language speech.

We employed Italian as the non-meaningful background stimuli with our Finnish-speaking readers, because phonetically Italian resembles Finnish. These two languages have similar consonant-vowel clusters, diphthongs, and double consonants; moreover, words typically end with a vowel in both languages. We ensured that the participants did not speak or understand Italian.

When studying effects of irrelevant speech on written language processing using the eye-tracking technique, the most likely index of disruption effects are the re-reading fixations that reflect efforts to reprocess and/or reactivate relevant parts of the text. As noted above, this was exactly what Cauchard et al. [2] observed in their eye-tracking study. Moreover, numerous studies have demonstrated that reinspective fixation time is a particularly sensitive index of syntactic reanalysis of sentences [33]. Moreover, a study [34] have demonstrated that readers retain relevant information by looking back to the text location that contains it.

### Method

#### Participants

Forty-two university students served as participants to fulfill a course requirement. All were native Finnish speakers, who did not understand or speak Italian. A written informed consent form was signed by each participant prior to the beginning of the experiment. None of the participants in Experiment 1 or in the subsequent experiments was a minor. Participants were also told that, if wished to do so, they could terminate the experiment at any time. The same procedure was applied to all subsequent experiments. Experiment 1 was administered to the participants by the second author. In order to anonymize the participants’ identity, in all data files the participants were identified using number codes.

#### Apparatus

Eye movements were collected by the EyeLink I eye-tracker manufactured by SR Research Ltd. (Canada). The eye-tracker is an infra-red video-based tracking system combined with hyperacuity image processing. There are two cameras mounted on a headband (one for each eye) including two infra-red LEDs for illuminating each eye. The cameras sample pupil location and pupil size at the rate of 250 Hz. Recording is monocular and is performed for the selected eye by placing the camera and the two infra-red light sources 4–6 cm away from the eye. The resolution of eye position is 15 seconds of arc and the spatial accuracy is better than 0.5 degrees. Head position with respect to the computer screen is tracked with the help of a head-tracking camera mounted on the centre of the headband at the level of the forehead. Four LEDs are attached to the corners of the computer screen, which are viewed by the head tracking camera, once the subject sits directly facing the screen. Possible head motion is detected as movements of the four LEDs and is compensated for on-line from the eye position records.

#### Materials

Text materials. Two types of target sentences, syntactically more complex and less complex sentences, were embedded in three texts, 10 complex and 10 less complex sentences in each text. One text was read in silence, one with Finnish background speech, and one with Italian background speech. All target sentences began with a noun phrase constituting the initial constituent of the main clause (either subject or object), followed by a center-embedded relative clause, and completed by the rest of the main clause. For the more complex sentences, the initial noun phrase was a clause object (it was marked with the so-called partitive case inflection), whereas for the less complex sentence it was a clause subject (it appeared in the nominative case, i.e., in the base-form with no case inflection). Matched pairs of sentences were created, which conveyed a highly similar meaning, but differed in syntactic complexity. An example of a sentence pair is given below with literal English translations.

**More complex**: *Pyramidialuetta*, *joka oli tärkeä osa kaupunkia*, *ihmiset kutsuivat nimellä Kuolleiden kaupunki*.

“The pyramid area that was an important part of the town people called the Town of the Dead.”

**Less complex**: *Pyramidialue*, *joka oli tärkeä osa kaupunkia*, *muodosti niin sanotun Kuolleiden kaupungin*.

“The pyramid area that was an important part of the town comprised the so-called Town of the Dead.”

The center-embedded relative clause was always identical in the two versions. In the less complex sentences, the structure of the main clause conformed to the default SVO word order in Finnish in that it was initiated with a clause subject, whereas the word order of the more complex sentences was syntactically marked in that they began with a clause object and the subject appeared only after the relative clause. The syntactic complexity in the object-fronted sentences is exacerbated by the fact that the discourse entity referred to by the initial noun phrase (i.e., *pyramidialuetta* in the above example) and by the relative pronoun (i.e., *joka* = that) is associated with two syntactic roles: the object of the main clause and the subject of the relative clause. For the less complex sentences, this dual-role feature does not exist, but the entity appears as the syntactic subject in both clauses.

The target sentence length was closely matched across the sentence pairs both as the number of words (the average was 12.0 words for both sentence types) and as the number of characters (the average was 92.9 and 94.2 for the less complex and more complex sentences, respectively). Each participant read only one version of each sentence pair. Two versions of each text were prepared, and the two members of each sentence pair appeared in different versions. The text versions were counterbalanced across participants so that each sentence was read with an equal number of times. The target sentences were spread across the texts so that they never appeared adjacent to each other.

The three texts in which the target sentences were embedded were entitled “The holy Ganges”, “The Egyptian pyramids”, and “The riddle of Stonehenge”. The texts were written with the help of encyclopaedias and other written sources. The length of the texts varied between 532 and 598 words. The participants read all three texts, one in each experimental condition. The texts were counterbalanced across the background speech conditions so that each text appeared in each condition an equal number of times. Also the order of the background speech conditions was counterbalanced across participants.

True-false statements. Twenty true-false statements were constructed for each text, one for each target sentence. The true statements were paraphrases of the target sentences, while the false statements contradicted some aspect of the target sentence. For each participant, of the probed sentences 10 sentences were presented in the text in OVS word order and 10 sentences in SVO word order. Response accuracy was not influenced by background condition or sentence type (*F*<1; see Table 1). This was true for the other experiments too; thus, these data are not presented for the subsequent experiments.

**Table 1:** Means and Standard Deviations (%) of the Accuracy in Responding to the True-False Statements Constructed for the Target Sentences, as a Function of Sentence Complexity and Background Condition (Silent, Italian, Finnish).

	Sentence Type				
	Complex		Less complex		
Background Condition	M	SD	M	SD	Grand mean
Silent	76	15	80	15	78
Italian	79	15	77	14	78
Finnish	75	14	74	12	75

Sound materials. The irrelevant speech was stored in a CD ROM disk and was played with the volume level of 80–85 dBA via two loud speakers that were placed on either side of the computer monitor. The Finnish speech comprised an excerpt of a novel, and the Italian speech was a collection of language course materials. Both speeches were delivered by a male voice. The speeches were made long enough to extend over the whole time the participants spent reading the text. In a short questionnaire given after reading, the participants reported irrelevant speech to feel moderately disruptive; Finnish background speech was assessed more disrupting that Italian background speech (the means were 3.81 and 2.40 using a 5-point scale; *t*(41) = 8.78, *p* < .001).

#### Procedure

Before the actual experiment, the eye-tracker was calibrated for each participant. Participants were instructed to read the texts to be able to answer true-false statements about the text contents. They were also told to ignore the background speech. Reading was self-paced with the restriction that returning to a previous page was prevented. Before a new text screen was presented, the participant had to look at a fixation point at the top-left corner of the monitor for an automatic correction of calibration. There were 6–7 lines of text per page. A short practice trial preceded the first text to adjust the participants to the eye-tracking equipment and to present the instructions. After each text, participants responded to 20 true-false statements. At the end of the session, they filled in a short questionnaire about their reading habits, and gave a rating of how much they were disturbed by the two types of background speech. The experimental session lasted for about one hour.

#### Dependent variables

Several eye fixation indices were employed as sentence processing measures. First, a distinction was made between first-pass and second-pass reading. First-pass reading consists of fixations made when the target sentence was initially read through, while second-pass reading comprises all look-back fixations that return to a target sentence from a subsequent sentence. Our definition for first-pass reading [35] departs somewhat from the criteria typically used in the eye movement literature. As the target sentences were typically preceded by other sentences on the same page, readers sometimes looked back to a previous sentence well before completing reading the target sentence. Therefore, the use of the standard first-pass measure, for which the inclusion of fixations is terminated whenever a fixation is made out of the target sentence, may give a somewhat misleading picture of the processing. Therefore, we adopted a first-pass measure that allows fixations to a previous sentence without terminating the target sentence’s first-pass reading, as long as there are at least two returning fixations to the target sentence that land further into the sentence than any of the fixations prior to backtracking.

Within our first-pass measure, we made a further distinction between progressive and rereading fixations. By definition, progressive fixations land on previously unread portions of the target sentence, while rereading fixations are fixations that land on target sentence regions that have already once been fixated. The distinction between progressive and rereading fixations was made by flagging the horizontal position of the fixation that has proceeded furthest in the given sentence. When the following fixation was positioned further into the sentence as the last flagged fixation, it was considered a progressive fixation; if not, it was considered a rereading fixation.

Progressive fixation time and rereading time is the summed duration of progressive versus rereading fixations. Rereading time is analogous to one proposed by Liversedge et al. [33] or the first-pass regression time of Van Gompel et al. [36], applied to whole sentences as targets. Finally, second-pass reading of sentences was examined by look-back fixation time. This measure sums up the fixations that land on the target sentence from a subsequent sentence after the first-pass reading of the sentences was terminated. In addition to the duration measures, we also computed comparable fixation frequency measures. However, as the results for the number of fixation measures were very similar to those for the duration measures, we report here only the duration measures.

#### Statistical analyses and experimental design

Repeated measures analyses of variance were computed with sentence type and background speech condition as within-participants variables. The dependent variables (all sentence-level variables) were first-pass fixation time, which was further divided into first-pass progressive fixation time and first-pass rereading time, and look-back fixation time. We provide the data for all four experiments in S1 Dataset.

### Results

#### First-pass reading

For the first-pass fixation time, the main effect of sentence type was significant, *F*(1, 41) = 9.47, *MSE* = 236995, *p* < .01, η_p_^2^ = .19; syntactically more complex sentences were read with longer first-pass fixation times than less complex sentences (a difference of 189 ms, see Table 2). The main effect of background condition was nonsignificant, *F*(2,40) = 2.25, *MSE* = 323220, *p*>.1, η_p_^2^ = .05. However, the quadratic trend proved significant, *F*(1,41) = 4.05, *MSE* = 292140, *p* = .05, η_p_^2^ = .09. It reflects the fact that first-pass fixation time was shorter in the Italian background condition than the other two conditions. Sentence type did not interact with background speech, *F*< 1.

**Table 2:** Means and Standard Deviations of the First-Pass Fixation Time (in ms) for the Target Sentence in Experiments 1–4, as a Function of Sentence Complexity and Background Condition.

	Sentence Type				
	Complex		Less complex		
Background condition	M	SD	M	SD	Grand mean
**Experiment 1**					
Silent	4343	1167	4134	910	4239
Italian	4183	899	4084	904	4134
Finnish	4448	1053	4189	891	4319
**Experiment 2**					
Silent	3708	937	3598	883	3653
Scrambled-Different	4153	1021	3929	1058	4041
Scrambled-Same	4156	857	3909	1026	4032
**Experiment 3**					
Silent	4161	1273	3915	1099	4038
Coherent	4271	1225	4242	1307	4257
Scrambled	4683	1681	4288	1432	4485
**Experiment 4**					
Silent	4416	1102	4205	1090	4311
Scrambled-Semantic	4682	1358	4575	1257	4629
Scrambled-Syntactic+Semantic	4679	1035	4512	1133	4596

As noted above, first-pass fixations were further divided into progressive and rereading fixations. Progressive fixation time did not show any significant effects (all *F*s<1.31; see Table 3). On the other hand, a reliable main effect of sentence type emerged in the rereading time, *F*(1, 41) = 12.31, *MSE* = 134644, *p* = .001, η_p_^2^ = .23; syntactically more complex sentences were read with longer rereading times than less complex sentences (see Table 4). The main effect of background speech was marginally significance, *F*(2,40) = 2.50, *MSE* = 154080, *p* = .09, η_p_^2^ = .06. This marginal effect reflects the tendency for the Italian background speech producing the shortest rereading fixation time. The interaction remained non-significant, *F*<1.

**Table 3:** Means and Standard Deviations of the First-Pass Progressive Fixation Time (in ms) for the Target Sentence in Experiments 1–4, as a Function of Sentence Complexity and Background Condition.

	Sentence Type				
	Complex		Less complex		
Background condition	M	SD	M	SD	Grand mean
**Experiment 1**					
Silent	3216	692	3179	639	3198
Italian	3116	524	3137	629	3127
Finnish	3213	673	3149	595	3181
**Experiment 2**					
Silent	2954	760	2915	660	2935
Scrambled-Different	3020	655	2960	719	2990
Scrambled-Same	2993	570	2969	720	2981
**Experiment 3**					
Silent	3194	875	3108	805	3151
Coherent	3201	836	3220	972	3210
Scrambled	3313	915	3229	940	3271
**Experiment 4**					
Silent	3251	713	3186	662	3219
Scrambled-Semantic	3327	783	3260	759	3294
Scrambled-Syntactic+Semantic	3292	616	3197	642	3245

**Table 4:** Means and Standard Deviations of the First-Pass Rereading Time (in ms) for the Target Sentences in Experiments 1–4, as a Function of Sentence Complexity and Background Condition.

	Sentence Type				
	Complex		Less complex		
Background condition	M	SD	M	SD	Grand mean
**Experiment 1**					
Silent	1127	734	955	589	1041
Italian	1067	662	947	542	1007
Finnish	1235	648	1040	537	1138
**Experiment 2**					
Silent	754	360	683	403	718
Scrambled-Different	1133	653	969	564	1051
Scrambled-Same	1163	647	940	555	1051
**Experiment 3**					
Silent	967	796	807	512	887
Coherent	1070	694	1023	656	1046
Scrambled	1370	1250	1059	843	1214
**Experiment 4**					
Silent	1165	650	1018	666	1092
Scrambled-Semantic	1355	923	1315	804	1335
Scrambled-Syntactic+Semantic	1387	811	1315	769	1351

To sum up the analyses of the first-pass reading, the observed syntactic complexity effect did not interact with the type of background speech, but it was comparable in size across the three conditions. There was a hint suggesting that non-meaningful speech would disrupt reading less than meaningful speech. Yet, meaningful speech did not differ from silence. The observed effects were due to rereading fixations, whereas progressive fixations were not reliable affected by the manipulations.

#### Look-back fixation time

In order to examine whether any effect may have appeared with some time lag, we analyzed the fixations that returned back to the target sentence from a subsequent sentence. However, these look-back fixations were not affected by the manipulated variables (all *F*s<1; see Table 5). As apparent from the huge standard deviations, some readers looked back in text quite extensively, while others looked back only minimally; for marked individual differences in the number of look-back fixations among competent adult readers, see [37].

**Table 5:** Means and Standard Deviations of the Look-Back Fixation Time (in ms) for the Target Sentences in Experiments 1–4, as a Function of Sentence Complexity and Background Condition.

	Sentence Type				
	Complex		Less complex		
Background condition	M	SD	M	SD	Grand mean
**Experiment 1**					
Silent	815	963	821	1212	818
Italian	759	802	737	785	748
Finnish	839	936	766	734	803
**Experiment 2**					
Silent	515	760	419	701	467
Scrambled-Different	535	1151	452	814	494
Scrambled-Same	515	920	560	957	538
**Experiment 3**					
Silent	405	614	405	517	405
Coherent	523	644	559	782	541
Scrambled	692	954	438	599	565
**Experiment 4**					
Silent	612	815	536	685	574
Scrambled-Semantic	580	698	515	519	548
Scrambled-Syntactic+Semantic	694	699	587	674	641

### Discussion

The results of Experiment 1 established a clear syntactic complexity effect in sentence processing during text reading. Additional first-pass fixation time was devoted to sentences in which word order in the main clause was changed from the default SVO order to a less common but acceptable OVS or OSV order. An added difficulty of the marked word-order sentences stems from the fact that the relative pronoun referring to the initial noun phrase of the main clause is syntactically incongruent with its referent; that is, the pronoun is the clause subject, whereas its referent occupies the role of syntactic object.

More importantly for the present study, we found no evidence for the interference in reading by background speech. We only found a tendency for the non-native language background speech producing less first-pass fixation time than the other two background speech conditions. The syntactic complexity effect was not found to be modulated by the presence of irrelevant background speech. The phonological loop model [15,17] predicts that the syntactic complexity effect in reading should have been more robust during irrelevant speech than during silence. However, the predicted interaction was clearly non-significant in all processing measures we employed. Thus, we were not able to find support for the view put forth by Gathercole and Baddeley [17] that phonological loop would particularly contribute to the processing of long, syntactically complex sentences. On the other hand, the results are generally consistent with the view of Sörqvist and Marsh [23], who posit that processing difficulty may shield against disruption by task-irrelevant stimuli.

The main outcome of Experiment 1 was the lack of interference in reading due to background speech. It appears exposure to non-native-language background speech or coherent native-language background speech does not disrupt the comprehension of center-embedded relative clause sentences. It is possible that a stronger manipulation is required to interfere with the online syntactic and semantic encoding of sentence information (see Gordon et al., 2001). Thus, in the subsequent experiments, we aimed to interfere more strongly with semantic and syntactic processing using anomalous background speech.

## Experiment 2

On the basis of the interference-by process account of auditory distraction of Marsh et al. (2008), it may be argued that disruption in reading comprehension due to irrelevant background speech is caused by the two information sources (written text and background speech) calling for and activating shared semantic representations and processes. In the Introduction, we sketched an analogous account for syntactic information. According to this account, shared syntactic representations and processes between the text to-be-read and the background speech would be responsible for the disruption effects in reading.

In Experiment 2, we interfered with semantic and syntactic processing by presenting during reading anomalous speech in the background. Anomalous background speech was produced by first scrambling the word order of a coherent text and then reading aloud the scrambled words in normal sentence intonation. As grammatical roles of clause constituents are denoted by case-marking in Finnish (there are 13 inflectional cases in active use), scrambling the word order of a coherent text results in the speech being absolutely “non-parsable” syntactically. Moreover, even though isolated words in scrambled speech as such are meaningful and comprehensible, their sequences are also semantically non-interpretable.

We created two types of scrambled speech, coined the Scrambled-Same and the Scrambled-Different condition. In the Scrambled-Same condition, we played in the background a scrambled version of the text the reader was currently reading. Thus, the words that appeared in the written text also appeared in a random order in the background speech. There is namely evidence that the effect of background speech on memory is greater when the irrelevant speech is semantically related to the to-be-remembered words [20,38]. For the Scrambled-Different condition, on the other hand, a scrambled version was created of a text dissimilar in content to the experimental texts. If similarity in semantic features between the two information sources is a pertinent factor in interference, the Scrambled-Same condition should be particularly disruptive. On the other hand, if syntactically anomalous background speech interferes with written sentence processing, the two anomalous background conditions should lead to disruption of similar magnitude. This is because they are syntactically equally anomalous. Similarly to Experiment 1, we assumed the disruption in reading to materialize particularly as increased number of rereading fixations done during the target sentence processing.

How plausible is it to assume that scrambled background speech will interfere with reading? A study [39] provided evidence that syntactically and semantically illegal background speech indeed disrupts language comprehension. It was observed that particularly syntactically illegal background speech produced a robust interference effect on correctly answering auditorily presented questions. It produced the most interference among the irrelevant speech conditions tested (semantically related, semantically unrelated, and non-word condition). The two semantic conditions produced more interference than the non-word condition.

Moreover, a neuroimaging study [40] provided evidence that scrambled speech spoken with normal intonation gains access to the syntactic module responsible for parsing normal sentences. It examined the cortical activation produced by auditorily presented normal and scrambled sentences that were output either with normal or list-like prosody. Relevant to the present study is the comparison between normal and scrambled sentences when they are presented with normal prosody. The study found that a dorsal region of the left anterior temporal lobe along the superior temporal gyrus responded both to syntactically structured sentences and to syntactically anomalous speech with sentence-like prosody. It is speculated that this region participates in the integration of prosodic cues with syntactic computations. What is relevant in the present context, the study found a brain region that responds equally strongly to normal speech and scrambled speech output with sentence-like prosody. Thus, the assumption that syntactically anomalous speech spoken with normal intonation gains access to the module responsible for sentence parsing seems neurally plausible.

Finally, more indirectly related to Experiment 2, a study [41] found that overhearing only a half of a phone conversation (“halfalogue”) disrupts processing in attentional tasks more than hearing both sides of the dialogue. This is presumably due to the halfalogue being less predictable in information content than a dialogue. Scrambled word salad used in Experiment 2 is another type of highly unpredictable speech. Thus, also from this perspective, it is likely to result in disruption in information processing, in our case in reading.

### Method

#### Participants

Thirty-six university students participated in the experiment to fulfill a course requirement. All were native Finnish speakers. None had participated in Experiment 1. The experiment was administered to the participants by a research assistant familiar with eye movement recording.

#### Apparatus

An EyeLink II eye-tracker was used to record readers’ eye fixation patterns. EyeLink II is an upgraded version of EyeLink I used in Experiment 1. The sampling rate was upgraded to 500 Hz.

#### Materials

The same texts were used as in Experiment 1. However, the background speech conditions were different. The silent condition was compared to two anomalous speech conditions, which were read by the same male speaker and played back to the participants via two speakers positioned on each side of the monitor. The average loudness level was adjusted to be analogous to the one used in Experiment 1. The background speech materials were created by scrambling the words of coherent texts and reading aloud these scrambled-word lists with an intonation mimicking that of coherent speech. To preserve the overall intonation pattern of coherent Finnish speech, the speaker segmented the words into “sentences” and adjusted his intonation accordingly. Two scrambled word lists were created. The Scrambled-Different condition was created by randomizing the order of words of a newspaper article on national politics. The Scrambled-Same condition was created by randomizing the order of words of the three experimental texts and presenting in the background that scrambled version of the text the participant was currently reading.

#### Procedure

The experimental procedure was identical to that of Experiment 1.

### Results

An analogous set of analyses was conducted as in Experiment 1 using univariate analyses of variance.

#### First-pass reading

The main effect of sentence type was highly significant, *F*(1,35) = 12.25, *MSE* = 165706, *p* = .001, η_p_^2^ = .26, in the first-pass fixation time (see Table 2). The syntactically more complex sentences were read with 194 ms longer first-pass fixation times than the less complex sentences. The effect is comparable in size to that observed in Experiment 1. The main effect of background speech proved also significant, *F*(2,70) = 12.24, *MSE* = 289219, *p* < .001, η_p_^2^ = .26; the two irrelevant speech conditions produced much longer first-pass fixation times than the silent condition (the difference is almost 400 ms), but the type of irrelevant speech did not seem to matter, as the difference between the two types of anomalous speech was only 9 ms. The Sentence Type x Background Speech interaction was non-significant, *F*<1.

When the first-pass fixations were further divided into progressive and rereading fixations, it became clear that the observed effects were primarily due to rereading fixations. In the first-pass progressive fixation time (see Table 3), there was only a suggestion for a main effect of sentence type, *F*(1,35) = 2.56, *MSE* = 35816, *p*>.1, η_p_^2^ = .07. On the other hand, the rereading fixation time (see Table 4) was affected both by sentence type, *F*(1,35) = 12.62, *MSE* = 99629, *p* = .001, η_p_^2^ = .27, and background speech, *F*(2,70) = 16.14, *MSE* = 164920, *p* < .001, η_p_^2^ = .32. The size of the syntactic complexity effect was 153 ms and that of the background speech effect 333 ms (the rereading fixation time was identical for the two types of anomalous background speech). The interaction remained non-significant, *F*<1. As the two scrambled speech conditions behaved highly similarly, we computed an average of the two and compared it to the silent condition in a 2x2 ANOVA. The interaction was statistically marginal, *F*(1,35) = 3.21, *MSE* = 42165, *p* = .08. The syntactic complexity effect in rereading fixation time was 71 ms in silence and 193 ms with the scrambled background speech.

In sum, the analyses of first-pass reading demonstrated a syntactic complexity effect, which was marginally bigger in size when reading was done with anomalous speech played in the background than in silence. Scrambled background speech reliably disrupted reading, but the disruption was comparable in size regardless of the overall semantic content of the scrambled speech. The observed effects were primarily due to first-pass rereading fixations.

#### Look-back fixations

Look-back fixation time was not reliably influenced by the manipulated factors, all *F*s<2 (see Table 5).

### Discussion

Experiment 2 replicated the syntactic complexity effect observed in Experiment 1 and many previous studies. More importantly for the present study, the two syntactically and semantically anomalous background speech conditions produced robust interference in reading. The first-pass fixation time on the target sentences was almost 400 ms longer in the two background speech conditions than in the silent reading condition. This effect was primarily a result of an increased rereading made during the first-pass reading through the target sentence, as the difference between the silent and the two background speech conditions was 333 ms in the rereading time.

The background speech effect did not differ in size between the two scrambled speech conditions. If anything, the effect was slightly bigger when the scrambled speech contained words that were semantically unrelated to the text to be read than when the scrambled speech was constructed of the same text the participants were reading. This finding is inconsistent with the view that there is a significant lexical-semantic component in the observed disruption effect, at least when it comes to disruption by shared overall semantic content. Instead, the data of Experiment 2 are more compatible with the view that the effect is more syntactic in nature. First, as the two background speech conditions are equally anomalous syntactically, they should be equally disruptive–an effect obtained in Experiment 2. Second, a statistically marginal tendency was observed in the rereading fixation time for a greater syntactic complexity effect for the target sentences in the scrambled speech condition (average of the two) than in silence. Although generally consistent with the syntactic account, the findings may also be explained by the semantic account. Syntactic complexity brings with it also semantic difficulty. Thus, scrambled speech may interfere with semantic processing without a need for the two information sources being similar in content. A further comparison of the semantic and syntactic accounts was carried out in Experiment 4.

Taken together the main findings of Experiment 1 and 2, it appears that meaningful, coherent speech does not notably disrupt the reading of long, syntactically complex sentences, but syntactically and semantically anomalous background speech significantly interferes with sentence processing. The finding that meaningful background speech does not bring about robust interference in reading is inconsistent with off-lines studies of reading comprehension reviewed in the Introduction. Thus, it is a surprising finding that needs to be replicated. Experiment 3 was conducted with this aim in mind.

## Experiment 3

The first goal of Experiment 3 was to re-examine whether it is indeed the case that coherent speech would not lead to marked disruption in reading, compared to silence, as observed in Experiment 1. The second aim of Experiment 3 was to directly compare disruption effects by meaningful speech (Experiment 1) to those of scrambled speech obtained in Experiment 2 (both semantically unrelated to the text to be read). Experiment 1 suggested that coherent irrelevant speech does not interfere markedly with sentence parsing, while Experiment 2 provided evidence for notable disruption in sentence processing due to anomalous background speech. In Experiment 3, we pitted these two background speech conditions against each other as a within-participants comparison. More robust interference by anomalous speech than coherent speech would be evidence supporting both the syntactic and semantic account. If the processing system makes attempts at processing syntactically and semantically anomalous speech, it should bring about interference in semantic and syntactic processing during reading.

### Method

#### Participants

Thirty-six university students participated in the experiment to fulfill a course requirement. All were native Finnish speakers. None had participated in Experiment 1 or 2. One participant had to be discarded due to poor calibration of the eye-tracker. The experiment was administered to the participants by the first author.

#### Apparatus

An EyeLink II eye-tracker was used to record readers’ eye fixation patterns. The sampling rate of 500 Hz was used.

#### Materials

The same texts were used as in Experiment 1 and 2. There were three background speech conditions: The silent condition was compared to two irrelevant speech conditions, scrambled speech and coherent speech. For the scrambled speech condition, the order of words of an unrelated text (about healthy nutrition) was randomized, and this scrambled-word list was read aloud with an intonation mimicking that of coherent speech. To preserve the overall intonation pattern of coherent Finnish speech, the speaker segmented the words into “sentences” and adjusted his intonation accordingly. For the coherent speech condition, another unrelated text (a globalization critique) was chosen. The scrambled word list and the coherent text were read by the same male speaker and played back to the participants via two speakers positioned on each side of the monitor. The average loudness level was adjusted to be analogous to the one used in Experiment 1 and 2.

After reading, participants were asked to rate, using a 5-point scale, how disturbing the two types of background speech were. They rated the scrambled speech somewhat more disturbing than the coherent speech, *t*(34) = 3.43, *p* < .01; the means were 3.49 and 2.97 for the scrambled and coherent speech, respectively (1 = not at all disturbing; 5 = highly disturbing). Surprisingly, only half of the participants noticed that the other background speech was anomalous.

#### Procedure

The experimental procedure was identical to that of Experiment 1 and 2.

### Results

An analogous set of ANOVAs as in Experiment 2 was computed for the target sentences.

#### First-pass reading

In the first pass fixation time, the main effect of sentence type was significant, *F*(1,34) = 7.58, *MSE* = 345408, *p* < .01, η_p_^2^ = .18. More complex target sentences resulted in 223 ms longer first-pass fixation times than less complex sentences (see Table 2). The main effect of background speech was also reliable, *F*(2,68) = 6.92, *MSE* = 505995, *p* < .01, η_p_^2^ = .17. The pairwise two-tailed *t* tests (all other *t* tests reported below are also two-tailed) showed that the silent condition differed reliably (*p* < .025) from both background speech conditions, whereas the two background conditions did not differ from each other. Moreover, the two main effects were qualified by a reliable interaction, *F*(2,68) = 3.04, *MSE* = 195821, *p* = .05, η_p_^2^ = .08. The syntactic complexity effect was largest in the scrambled background speech condition (395 ms) and negligible in the coherent background speech condition (29 ms); in the silent condition the effect size was 246 ms. In pairwise comparisons of the three background speech conditions, the two-way interaction only emerged significant when coherent and scrambled conditions were compared to each other, *F*(1,34) = 5.26, *MSE* = 223494, *p* < .05.

The first-pass fixation time was again broken down to progressive and rereading fixation time. The progressive fixation time demonstrated an almost significant syntactic complexity effect, *F*(1,34) = 3.78, *MSE* = 35569, *p* = .06, η_p_^2^ = .10, and a reliable background speech effect, *F*(2,68) = 3.35, *MSE* = 75386, *p* < .05, η_p_^2^ = .09. A pairwise *t* test showed that the silent condition produced significantly shorter (*p* < .025) progressive fixation times than the scrambled background speech condition (see Table 3).

In the first-pass rereading time, both the main effect of sentence type, *F*(1,34) = 6.13, *MSE* = 255406, *p* < .02, η_p_^2^ = .15, and the main effect of background speech, *F*(2,68) = 5.56, *MSE* = 336473, *p* < .02, η_p_^2^ = .14, proved significant. Pairwise *t* tests showed that the silent condition differed from the two background speech conditions (p < .02), but the background speech conditions did not differ from each other (see Table 4). The interaction was nearly significant, *F*(2,68) = 2.76, *MSE* = 111182, *p* = .07, η_p_^2^ = .08. The scrambled condition produced the greatest syntactic complexity effect (321 ms) and the coherent condition the smallest complexity effect (47 ms). In pairwise comparisons of the three background speech conditions, it appeared that the syntactic complexity effect tended to be greater in the scrambled speech condition than either in the coherent speech, *F*(1,34) = 3.92, *MSE* = 155395, *p* = .06, or in the silent condition, *F*(1,34) = 2.97, *MSE* = 67461, *p* = .09.

The marginal interaction between syntactic complexity and background speech (silent vs. scrambled) compares favorably with an analogous marginal effect in Experiment 2. In order to increase statistical power, we pooled the data of these two experiments. In the pooled analysis, the interaction proved significant, *F*(1,69) = 6.09, *MSE* = 54630, *p* < .02, η_p_^2^ = .08. The complexity effect in rereading fixation time across the two experiments was 115 ms in silence and 252 ms when scrambled speech was played in the background.

#### Look-back fixations

Look-back fixation time was marginally influenced by background speech, *F*(2,68) = 2.76, *MSE* = 190066, *p* = .07, η_p_^2^ = .08. Pairwise *t* test showed that the silent condition differed significantly from the scrambled speech condition (*p* = .05) and marginally (*p* = .08) from the coherent background speech (see Table 5). This main effect was qualified by a Sentence Type x Background Speech interaction, *F*(2,68) = 4.32, *MSE* = 101344, *p* < .02, η_p_^2^ = .11. To break down the interaction, the syntactic complexity effect was tested in pairwise comparisons of the three background conditions. These analyses revealed that the interaction was significant both when the scrambled speech condition was compared to the silent condition, *F*(1,34) = 8.40, *MSE* = 67267, *p* < .01, and to the coherent speech condition, *F*(1,34) = 5.40, *MSE* = 136235, *p* = .03. As is apparent from Table 5, only the scrambled speech condition demonstrated a syntactic complexity effect.

### Discussion

We replicated the results of Experiment 2 by demonstrating that anomalous background speech significantly interferes with reading long, syntactically complex sentences. It is a robust effect, as the difference between the silent condition and the scrambled speech condition was consistently observed in all our eye fixation measures. Unlike in Experiment 1, we now obtained clear evidence that also coherent irrelevant speech disrupts written sentence processing. It is possible that difference in the voice characteristics may be at stake here. Although in both experiments the coherent speech was produced by a male speaker, the speaker in Experiment 1 had a more monotonous and peaceful voice than the speaker in Experiment 3. At this point this is only speculative but certainly worth studying in the future. Generally in line with the above speculation, prior studies of irrelevant speech effects on serial recall have demonstrated a greater disruption effect when the irrelevant speech involves phonological variability than when the same utterance is constantly repeated in the speech stream [42–43].

Based on the semantic and syntactic accounts, we also predicted anomalous speech to interfere more with reading than coherent, meaningful speech. In line with this prediction, the sentence complexity effect in reading was greater in the scrambled speech than in the coherent speech condition. This became apparent both in the first-pass and look-back fixation time of the target sentences. Anomalous background speech also produced a more sizeable sentence complexity effect in reading than the silent condition. This was seen in the rereading fixation time (particularly when the data from Experiment 2 and 3 were pooled) and look-back fixation time. These data suggest that anomalous background speech notably interferes with on-line processing of long, syntactically complex sentences. This may be interpreted as support for both the semantic and syntactic account, as the interfering scrambled speech was anomalous both semantically and syntactically.

We asked our participants after reading whether they noticed anything bizarre in either of the background speeches. Surprisingly, only half of the readers admitted doing so. Many of those who did not notice anything bizarre were very surprised to hear about the anomalous speech condition. We conducted post-hoc analyses to see whether any of the observed effects would be different in size between the readers who did or did not notice the bizarreness of the anomalous background speech. However, we found no suggestion in the data to support the view that the awareness of anomaly would have produced any discernible effects in written sentence processing (the details of the analyses are not reported). This suggests that, in order to materialize, disruption effects due to anomalous background speech do not need to be accompanied by conscious awareness of the nature of the interfering irrelevant speech.

## Experiment 4

As regards the nature of the effect of anomalous background speech in reading, the data of Experiment 2 and 3 can be accommodated either by the syntactic or the semantic account, as long as the emphasis is on shared processing and not on shared representations between the two information sources. This is because Experiment 2 obtained no evidence for the view that similarity in general semantic content between the two sources of information would exacerbate the disruption effect in reading. However, on the basis of Experiment 2 and 3 it is not possible to tease apart the relative contribution of the semantic and syntactic component, as the scrambled speech used was anomalous both syntactically and semantically. The random word salad did not conform to the syntactic rules of the language, neither did it express any sensible meanings, but rather strange combinations of words that may sometimes appear humorous. Experiment 4 was designed to help tease apart the effect of the two types of anomaly. This was done by testing disruption effects in reading for two types of scrambled speech: one that is both syntactically and semantically anomalous (similarly to Experiment 2 and 3) and another that is semantically anomalous but syntactically correct. The latter condition was created by first randomizing the order of words of a coherent text and then editing the word list in order to construct syntactically correct but semantically anomalous sentences. If syntactic anomaly is particularly disruptive, we should then find more robust interference effects for scrambled speech that is both syntactically and semantically anomalous than for scrambled speech that is only semantically anomalous.

### Method

#### Participants

Thirty-six university students, all native speakers of Finnish, participated in the experiment to fulfill a course requirement. None had participated in any of the previous experiments. The experiment was administered to the participants by the first author.

#### Apparatus

An EyeLink II eye-tracker was used to record readers’ eye fixation patterns. The sampling rate of 500 Hz was used.

#### Materials

The same texts were used as in the previous experiments. There were three background speech conditions: The silent condition was compared to two irrelevant speech conditions, both of which contained scrambled speech. The other scrambled speech condition was the same as used in Experiment 3. For this scrambled speech condition, the order of words of an unrelated text (about healthy nutrition) was randomized, and this scrambled-word list was read aloud with an intonation mimicking that of coherent speech. This condition is called Scrambled-Syntactic+Semantic, as it violated the syntactic structure of sentences and it was also semantically anomalous. For the other scrambled speech condition, called Scrambled-Semantic, the syntactic structure of sentences was correct but the sentences were semantically anomalous. The Scrambled-Semantic condition was created by randomizing the order of words of a coherent text dealing with sports and health; thus, it was semantically unrelated to any of the texts to be read. The word list was edited to make syntactically correct but semantically anomalous sentences. This was done by changing the inflectional endings of nouns and adjectives and, if required, changing the word order and the verb forms. Similarly to Experiment 3, in order to preserve the overall intonation pattern of coherent Finnish speech, in both conditions the speaker segmented the words into “sentences” and adjusted his intonation accordingly. The scrambled speech was produced by the same male speaker and played back to the participants via two speakers positioned on each side of the monitor. The average loudness level was adjusted to be analogous to the one used in the previous experiments.

After reading, participants were asked to rate, using a 5-point scale, how disturbing the two types of background speech were. They rated the Scrambled-Syntactic+Semantic condition somewhat more disturbing than the Scrambled-Semantic condition, *t*(35) = 2.28, *p* < .05; the means were 3.36 and 2.97 for the Scrambled-Syntactic+Semantic and the Scrambled-Semantic condition, respectively. We also asked after reading all texts whether they noticed anything bizarre in the background speech. Twenty out of 36 participants (56%) reported noticing some bizarreness.

#### Procedure

The experimental procedure was identical to that of the previous experiments.

### Results

An analogous set of ANOVAs as in Experiment 2 and 3 was computed for the target sentences.

#### First-pass reading

First pass fixation time revealed a significant main effect of sentence type, *F*(1,35) = 7.41, *MSE* = 191228, *p* = .01, η_p_^2^ = .18 (see Table 2). Syntactically complex sentences were read with 161 ms longer first-pass fixation times than less complex sentences. The main effect of background speech also reached significance, *F*(1,35) = 4.13, *MSE* = 532964, *p* = .02, η_p_^2^ = .11. An analysis of Helmert contrasts revealed that the silent condition produced shorter first-pass fixation times than the two scrambled-speech conditions, *F*(1,35) = 10.04, p < .01, which did not differ from each other, *F*<1. The interaction was non-significant, *F*<1.

The first-pass fixation time was again broken down to progressive and rereading fixation time. The only significant effect observed in progressive fixation time was the main effect of sentence type, *F*(1,35) = 11.54, *MSE* = 26685, *p* < .01, η_p_^2^ = .25; complex sentences received 76 ms more progressive fixation time than less complex sentences (see Table 3). In rereading fixation time, the main effect of background speech was significant, *F*(1,35) = 5.55, *MSE* = 274331, *p* < .01, η_p_^2^ = .14, whereas the main effect of sentence type failed to reach significance, *F*(1,35) = 2.74, *MSE* = 147176, *p* = .11, η_p_^2^ = .07. The silent condition produced shorter rereading fixations times than the two scrambled speech conditions, *F*(1,35) = 14.21, *p* = .001, which did not differ from each other, *F*<1 (see Table 4). The interaction was non-significant, *F*<1.

#### Look-back fixations

For look-back fixation time (see Table 5), all effects remained non-significant, *F*< 2.81.

### Discussion

Experiment 4 replicated some of the results of Experiment 2 and 3 by demonstrating that reading is disrupted by scrambled background speech. Participants needed to reread the target sentences for longer time during first-pass reading when scrambled speech was played in the background than when no background speech was present. The new aspect of Experiment 4 was to examine whether scrambled speech that is both syntactically and semantically anomalous would produce more disruption in reading than scrambled speech that is only semantically anomalous. Experiment 4 found no evidence supporting the view that syntactic anomaly would exacerbate the interference effect. This became apparent in the two scrambled speech conditions producing a disruption effect of similar size. In the General Discussion, we discuss in more detail the implication of this result in relation to other key findings observed in the present study.

**Fig 1:**
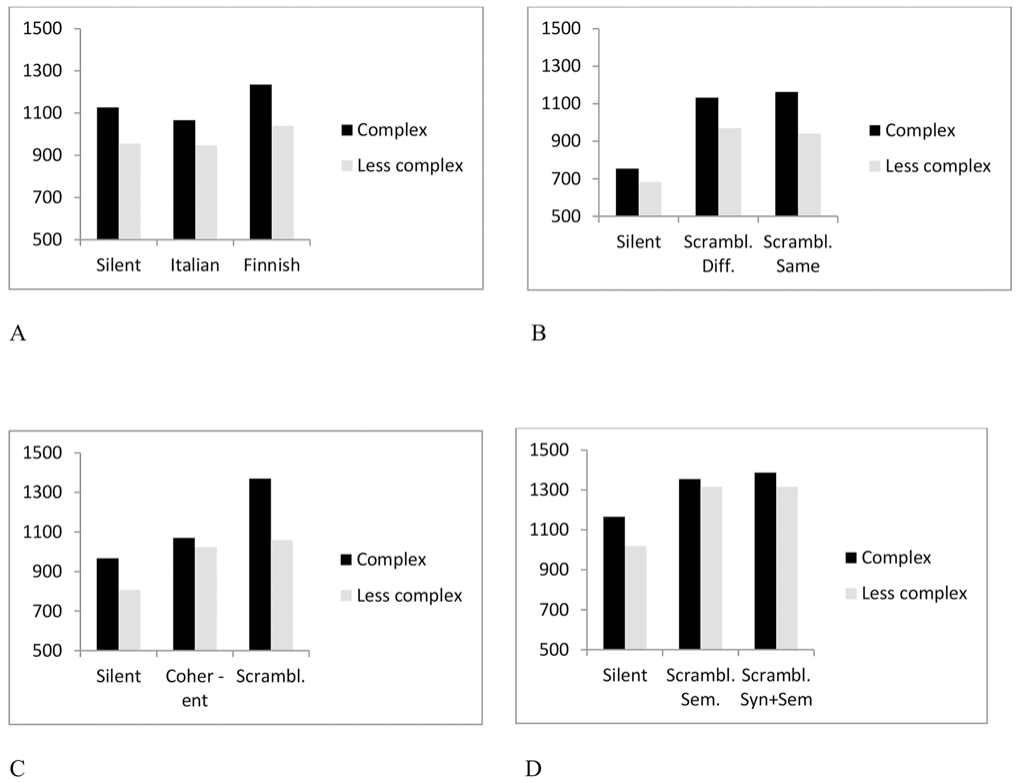
Rereading fixation time during first-pass reading of the target sentences, as a function of syntactic complexity and type of background speech. (A) Experiment 1. (B) Experiment 2. (C) Experiment 3. (D) Experiment 4.

The results of Experiment 4 depart from those of Experiment 2 and 3 by not providing evidence for the view that scrambled background speech would exacerbate the effect of syntactic complexity in sentence reading. Such an effect was observed in the pooled analysis of the rereading time in Experiment 2 and 3. What appears to be at stake here is that the participants of Experiment 4 reread the target sentences overall to a greater extent than those of Experiment 2 and 3 (see Fig 1). This is particularly noticeable for the scrambled speech conditions, where also the less complex target sentences were often partly reread. This diminished the difference in rereading time between the syntactically complex and less complex target sentences, leading to a non-existent Background Speech x Syntactic Complexity interaction. It is understandable given that even the less complex target sentences, containing a center-embedded relative clause, are syntactically quite complex.

## General Discussion

The present study was conducted to examine disruption effects in reading long, syntactically complex sentences due to different types of background speech played while participants were engaged in reading expository texts. Readers were asked to ignore the background speech and concentrate only on reading. Effects of background speech were investigated by registering readers’ eye movement patterns for a selected set of sentences embedded in relatively long texts. The target sentences were center-embedded relative clause sentences. For a half of the target sentences, syntactic complexity was further increased by changing in the main clause the word order from the default order (SVO) to a marked and more infrequent order (OSV or OVS).

The key findings of the present study are summarized in Fig 1, which depicts the results for the time spent rereading the target sentences during first-pass reading. Rereading time was the measure where the disruption effects due to background speech appeared most strongly and consistently. The main findings are the following: (1) Foreign language background speech (Italian) did not interfere with reading (see Fig 1A). (2) Anomalous background speech (the order of words of coherent speech was randomized and this scrambled list of words was presented auditorily with an intonation mimicking normal speech) produced marked disruption effects in written sentence processing (see Fig 1B, 1C and 1D). (3) Anomalous background speech produced more interference in reading than meaningful, coherent background speech. This was primarily seen in reading syntactically complex sentences (see Fig 1C). (4) The semantic relatedness of the scrambled word list to the to-be-processed written text did not lead to more severe disruption in reading than semantically unrelated scrambled speech (see Fig 1B). (5) Disruption effects in reading due to scrambled speech were equally robust when the scrambled speech was both semantically and syntactically anomalous than when it was only semantically anomalous (see Fig 1D).

As noted above, the disruption effects in processing due to background speech were observed in the rereading fixations (see also [2]) made during the first-pass reading of the target sentences, that is, before proceeding to the following sentence. Rereading fixations are known to index local comprehension difficulty and efforts in resolving such comprehension difficulties by reprocessing the text segments that have caused the comprehension difficulty. In the present study, this became apparent in the processing of long, syntactically complex sentences. Rereading fixations may also be used reactivate in the reader’s working memory relevant text information that may have been temporarily lost [34]. In the present study, this may frequently have happened in a situation where continuous speech is heard in the background during reading. Background speech may override the evolving mental representations of the text contents, which will then be reactivated by rereading the relevant text segments.

What may be the nature of the mental representations disrupted by background speech? In the Introduction, we outlined three alternative accounts regarding the nature of the disruption effects, the phonological, semantic and syntactic account. The underlying idea in all of them is that online text comprehension involves mental representations and/or processes that are automatically activated also by the task-irrelevant background speech. The shared representations and/or processes between the two information sources then interfere with the task performance, especially when task demands are high, as is the case in reading long, syntactically complex sentences.

The pattern of results does not provide support for the view that the disruption effects would be phonological in nature, as assumed by the working memory model of Baddeley [15]. According to the model, all speech-like sounds should interfere with written language comprehension, as they gain automatic access to the phonological short-term store that is assumed to be utilized in language comprehension. The interference is assumed to be particularly pronounced for long, syntactically complex sentences [17]. The evidence inconsistent with the phonological account is as follows. First, we found no evidence that foreign language background speech (phonetically similar to the readers’ native language) would interfere with reading long, syntactically complex sentences. As pointed out above, according to the model all irrelevant speech input should interfere with reading. Second, Background Speech x Syntactic Complexity interactions are problematic for a pure phonological account. Such an interaction was obtained in Experiment 3. This is problematic since coherent and anomalous background speech were phonologically identical in that they both conformed to the normal speech intonation and should thus have produced interference effects of similar size.

Researchers [6,20,21] have proposed a semantic account to explain interference effects in reading comprehension due to background speech. They proposed that reading comprehension is disrupted by irrelevant background stimuli when the distracting stimuli call upon the same semantic processing mechanisms and representations than the primary task. The finding that native-language background speech is more disruptive than foreign-language background speech (Experiment 1) is consistent with the semantic account. So is the finding that semantic anomaly of background speech was equally disruptive than semantically and syntactically anomalous background speech (Experiment 4). On the other hand, Experiment 2 provided evidence that is not completely in line with it. Scrambled speech created from the to-be-read text was found to be no more disruptive than scrambled speech created from a text different in semantic content with the to-be-read text. This finding suggests that similar semantic contents between the text to-be-read and the background speech is not crucial for producing interference in reading. However, this does not rule out the notion that the disruption effects are due to the two information sources both calling for semantic processing. In other words, it is not the shared semantic representations but shared semantic processing that may be at stake. Underlying this notion is the idea that background speech activates semantic processing even when it is ignored as irrelevant to the task at hand. Indeed, according to the interference-by-process account [20,21], performance in semantic memory tasks is impaired by auditory distraction due to the similarity of process activated by the task-relevant and task-irrelevant information, not due to similarity in content.

The syntactic account was theoretically derived from the SSIR model of verbal working memory [22]. The model assumes an independent syntactic module with its own resource pool. On the assumption (not explicitly stated in the theory) that this sentence-interpretation module attempts to parse all available linguistic input (including to-be-ignored irrelevant speech), it would unavoidably run into difficulties in attempting to parse syntactically anomalous background speech. These difficulties would in turn be reflected in simultaneous reading of syntactically complex sentences. Robust disruption effects in reading observed for anomalous background speech (Experiment 2 and 3) are generally consistent with this account. Moreover, the finding obtained in Experiment 3 that the disruption effect by anomalous speech was particularly robust for reading syntactically more complex sentences accords with the syntactic account. Finally, the finding that semantic relatedness between the words comprising the anomalous speech and those of the written text did not exacerbate the disruption effects in sentence processing (Experiment 2) is generally in line with the syntactic account. However, there is one puzzling result for the syntactic account. Semantically anomalous but syntactically correct background speech produced equal amount of interference for background speech that was both semantically and syntactically anomalous. If the interference is predominantly syntactic in nature, the presence of syntactic anomaly should have magnified the size of the interference effect.

To conclude the above discussion concerning the nature of the observed disruption effect in reading due to task-irrelevant background speech, we propose that the pattern of results obtained in the present study is best explained by a semantic account that stresses the importance of similarity in semantic processing, but not similarity in semantic content, between the reading task and background speech [20,21]. In other words, irrelevant background speech automatically activates semantic processing which then disrupts semantic processing of the text. The claim that the activation is automatic is supported by the finding that the interference effects in reading were similar in size between the participants who noticed or did not notice the anomaly of the scrambled background speech. Needless to say, further evidence will be needed to support our claims.

Yet another possibility is offered by the attentional capture account [44–46]. It is possible argue that anomalous background speech captures periodically readers’ attention and thus temporarily distracts the performance of the actual reading task. It may be further argued that all anomaly, be it semantic and/or syntactic in nature, has the capacity of capturing the reader’s attention. Yet, it should be noted that the present study does not provide direct evidence for or against this account.

Before closing, we would like to point to the potential fruitfulness of the interference paradigm in studying online language comprehension. By manipulating the nature and timing of the irrelevant background speech it is possible to discern what processes and representations are active in any given time during language comprehension. Inhoff et al. [47] have designed an attractive paradigm for controlling the timing of the background speech in relation to the text to-be-read. They examined phonological processing of words during reading by presenting auditorily a word that was identical, phonologically similar or dissimilar to the target word. By employing the boundary paradigm [48], auditory presentation of words was made contingent on the time when a fixation started on the target word. To study interference effects not limited to individual words, a more global manipulation of the irrelevant speech, as done in the present study, may be preferable.

## Supporting Information

S1 Dataset The file contains the data of all four experiments in different worksheets. (XLS)
